# Primary Cranial Vault Non-Hodgkin’s Lymphoma Mimicking Meningioma With Positive Angiography

**DOI:** 10.7759/cureus.8856

**Published:** 2020-06-27

**Authors:** John W Kiessling, Eric Whitney, Alessandra Cathel, Yasir R Khan, Deependra Mahato

**Affiliations:** 1 Neurosurgery, Desert Regional Medical Center, Palm Springs, USA

**Keywords:** neurosurgery, primary lymphoma, meningioma, chemotherapy, non-hodgkin's lymphoma

## Abstract

Primary non-Hodgkin’s lymphoma of the bone remains an uncommon presentation of non-Hodgkin’s lymphoma. Primary lymphoma of the cranial vault is exceptionally rare. Here, we present a 62-year-old immunocompetent male presenting with the rapid growth of a left parietal scalp lesion and new-onset seizure. In addition to his imaging, which showed an extracranial, cranial, and intracranial mass with bony destruction, sagittal sinus involvement, and parenchymal invasion, his diagnostic angiogram demonstrated extensive vascular supply from both the right and left external carotid branches. Intraoperatively, we confirmed a frank invasion of the posterior sagittal sinus. After subtotal resection followed by R-CHOP (rituximab, cyclophosphamide, doxorubicin, vincristine, and prednisone therapy, the patient continues to be disease-free at the 10-month follow-up. We report here a case of primary cranial vault lymphoma that very closely mimicked meningioma in many ways, with positive angiography and intraoperatively confirmed venous sinus invasion.

## Introduction

Primary non-Hodgkin’s lymphoma (NHL) of the bone remains an uncommon presentation of NHL, accounting for 0.3% to 5% of all NHL [1-5]. Primary malignant lymphoma of the bone is defined as “a solitary mass lesion with no evidence of disease at other sites and no systemic dissemination within 6 months of the detection of the tumor” [2-4,6]. Amongst these, a primary NHL of the cranial vault (PCVL) remains extremely rare, with less than 50 reported in the medical literature [6-8]. We describe a case of PCVL that closely mimicked a meningioma in an immunocompetent male that presented with extracranial, cranial, and extra- and intra-axial components, a high degree of vascularity, as well as sagittal sinus involvement.

## Case presentation

History and examination

A 62-year-old male on vacation presented to our emergency department after suffering a new, left-sided, simple partial seizure that became a generalized clonic seizure. He had been aware of a right parietal scalp mass that had rapidly progressed in size from a small nodule to approximately grapefruit-sized in four months. After resolution of his post-ictal state, he initially had upper and lower extremity paresis, which had resolved at presentation. Before his seizure, he had been completely asymptomatic other than noticing the physical features of the growing mass. He denied any previous neurological and constitutional symptoms, including night sweats, fever, chills, abnormal weight loss, or inguinal, axillary, or otherwise lymphadenopathy.

**Figure 1:**
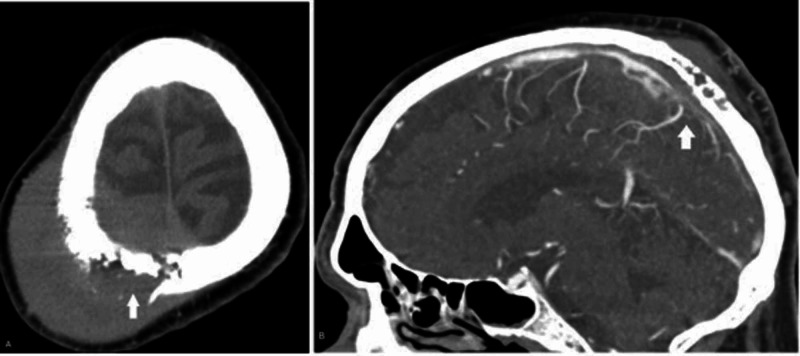
A. Axial CT head demonstrating a large subcutaneous mass with infiltration of the cranial vault and bony destruction. B. Sagittal CTA demonstrating bony destruction and superior sagittal sinus involvement. CT: computed tomography; CTA: CT angiography

On examination, the patient was alert, oriented, and without focal neurological deficits. No lymphadenopathy, splenomegaly, and hepatomegaly were appreciated. A roughly grapefruit-sized, hard, immobile, nontender right parietal mass under the normal-appearing scalp was appreciated. Computed tomography (CT) of the brain demonstrated a large subcutaneous mass with osteolytic infiltration of the right parieto-occipital bone with intracranial extension measuring 7.5 x 5 x 9.6cm. The mass was abutting the superior sagittal sinus (SSS). Bone windows showed frank bony destruction of the calvarium (Figure 1).

Due to the proximity and appearance of extension around the SSS, CT angiography (CTA) of the head and neck was obtained, which noted vascularity within the mass and the appearance of invasion into the posterior segment of the SSS.

**Figure 2:**
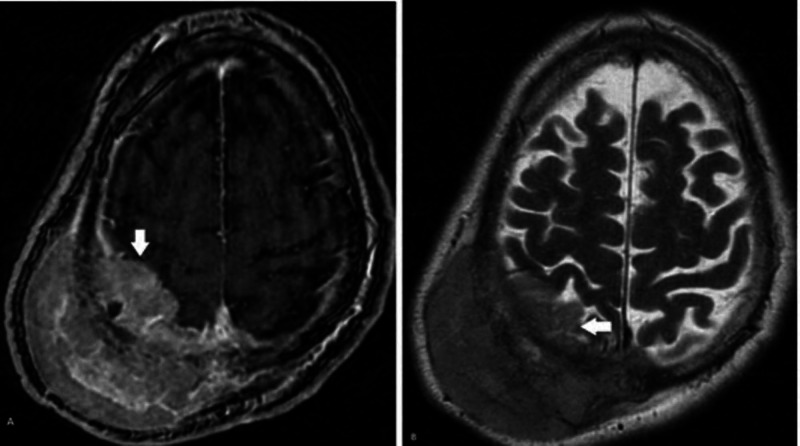
MRI brain with and without contrast demonstrating a somewhat heterogeneously enhancing mass with dural involvement and mild associated parenchymal edema A. axial post-contrast T1-weighted image, B. axial T2-weighted image MRI: magnetic resonance imaging

Magnetic resonance imaging (MRI) of the brain with and without contrast was obtained and showed a large right parietal mass with extension through the calvarium, crossing midline with the involvement of the posterior sagittal sinus, mass effect on the parietal lobe, and evidence of parenchymal invasion with associated cerebral edema. Also noted was extensive dural involvement and extension, consistent with possible atypical or malignant meningioma (Figure 2).

**Figure 3:**
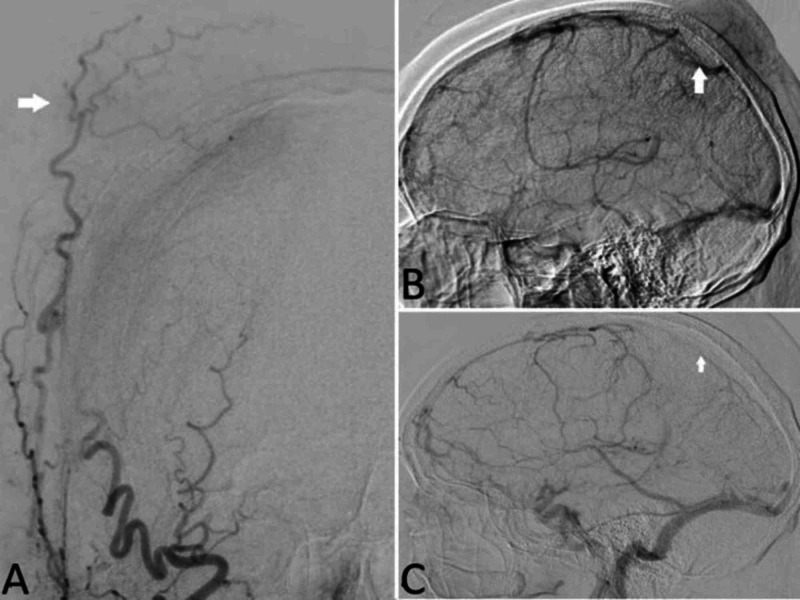
Angiography A. AP - selective angiography of the right ECA showing late arterial and capillary phase with mild tumor blush. B. Lateral-selective angiography of the right CCA showing the venous phase showing significantly reduced sagittal sinus flow. C. Lateral angiography in the venous phase showing significantly reduced sagittal sinus flow. AP: Anterior-Posterior; ECA: External Carotid Artery; CCA: Common Carotid Artery

Angiography was performed demonstrating the right parietal branch of the superficial temporal artery, as well as the occipital artery, appeared to supply the scalp portion of the mass. In addition, the distal parietal branch of the right middle meningeal artery (MMA) appeared to also supply the mass. Significant stenosis in the posterior third of the SSS but with continued patency was illustrated (Figure 3).

The patient was medically stabilized, started on levetiracetam and decadron, and a complete metastatic workup with contrasted CT of the chest/abdomen/pelvis was performed, which was unremarkable. Upon work-up completion, the patient elected to return home for any treatment and/or intervention. However, he re-presented within 24 hours with another generalized clonic seizure and now had persistent left upper and lower extremity paresis, continued left lower extremity clonic movements of which he had gross neglect, prolonged post-ictal period, and continued numbness and tingling of the left upper extremity. His levetiracetam was increased and valproic acid was added, but after several days, he continued to have intermittent simple partial seizures involving the left upper and lower extremity and significant left-sided paresis. Given his now progressive neurological decline, the patient was taken for right frontal-parietal decompressive craniectomy and resection of the mass.

Operation

**Figure 4:**
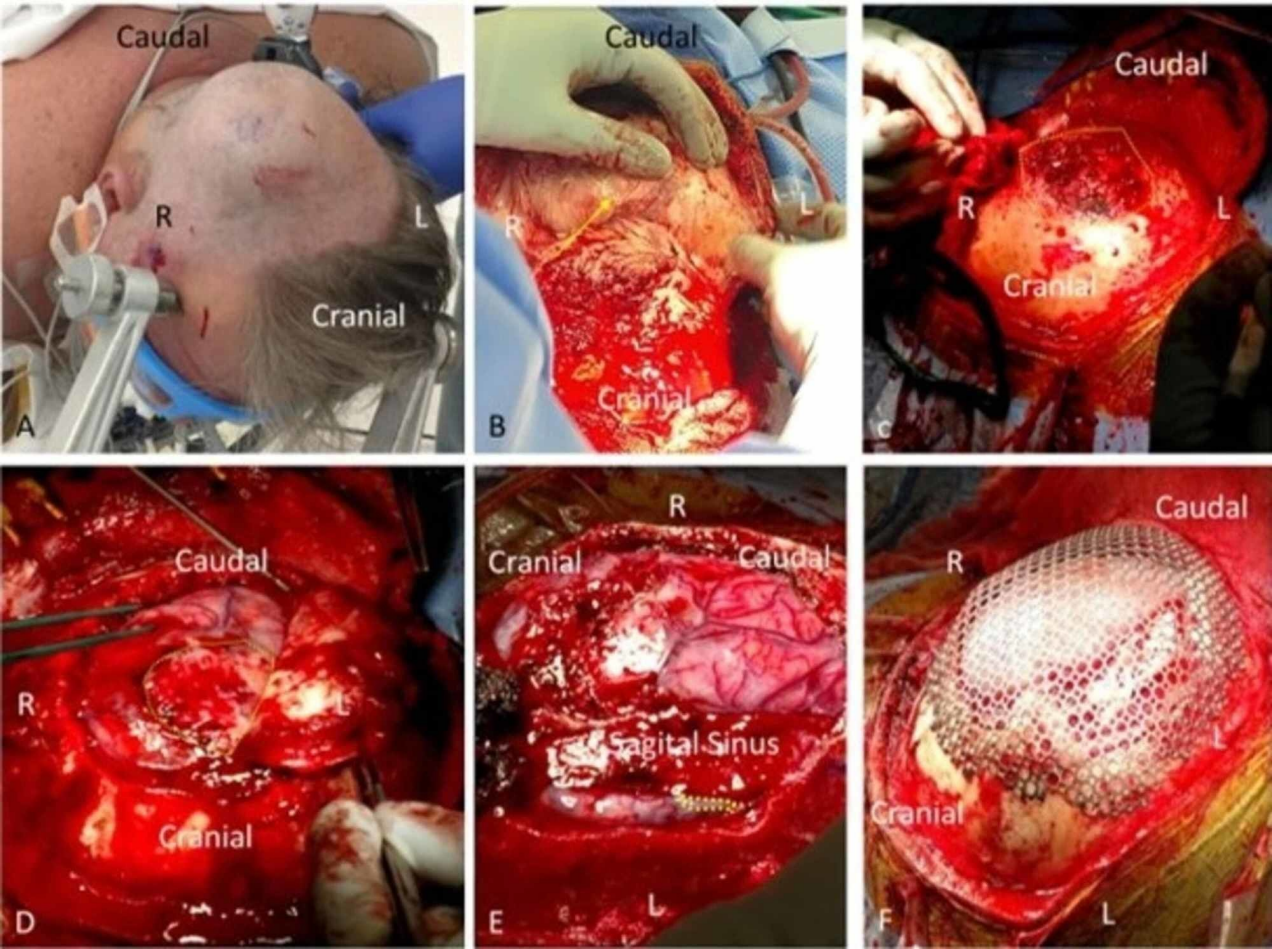
Intraoperative photos A: Gross appearance prior to incision. B: Highly vascular mass after incision and reflection of the scalp showing a large subcutaneous mass with mass coming from the calvarium. C: After superficial removal of the extracranial portion, the invasion through the calvarium can be appreciated. D: The en plaque presence over the motor and sensory gyri. The dura has been resected as much as safely possible and the portion involved and invading the sagittal sinus medially is being attempted to be dissected free. E: Demonstrates the extensive venous collateralization and engorgement due to the sinus stenosis and invasion. The tumor plaque continues to be evident and the superior sagittal sinus is skeletonized as much as possible. F: Titanium cranioplasty

Intraoperatively, a large, highly vascular, firm, dense mass was encountered that had effectively destroyed a large portion of the involved calvarium. On careful dissection, extensive dural involvement was found, with the extension of the mass through the dura, forming a plaque upon the right motor and sensory cortices with intraparenchymal invasion. The mass was also found to have invaded the SSS, which was skeletonized and debulked as much as possible. Ultimately, the patient received a subtotal resection with titanium cranioplasty (Figure 4).

Pathology

Postoperatively, initial pathology was reported as a possible World Health Organization (WHO) grade III meningioma. Stains on permanent showed positive CD45, positive BCL2, positive CD20, negative EMA, negative GFAP, and Ki-67 of 50%. Final pathology returned as diffuse large B-cell lymphoma (DLBCL) with fluorescence in situ hybridization (FISH) showing a 68% loss of BCL6. Additional cerebrospinal fluid (CSF) analysis was negative for any signs of atypical lymphocytes or lymphoid neoplasm.

Postoperative course and treatment

The patient underwent two cycles, one inpatient and one outpatient, of R-CHOP (rituximab, cyclophosphamide, doxorubicin hydrochloride, vincristine, prednisolone) chemotherapy as well as two rounds of intravenous (IV) rituximab and methotrexate for the noted intraoperative parenchymal invasion. While cerebrospinal fluid (CSF) studies were pending, he received a single round of intrathecal cytarabine given the dural invasion into and through the leptomeningeal space. After multiple CSF analyses demonstrated no abnormality, no further intrathecal therapy was administered. At discharge, he was neurologically intact, no further seizures, and at the time of this article, has completed his treatment and remains disease-free 10 months postoperatively.

## Discussion

Primary lymphoma of the cranial vault remains an extremely rare disease [5-8]. The review by El Asri and colleagues (2012) found 39 reported cases of patients with PCVL [6]. As with any primary bone lymphoma, those diagnosed must go at least six months after diagnosis without any evidence of distal lesions [1-4]. Within the reported literature, the most common pathology reported with PCVL is of DLBCL, ranging from 39% to 77% [6,8]. Primary dural lymphoma (PDL) is reported as a separate and distinct entity from PCVL. It is believed to arise primarily from the dura and its predominant pathology is of lower grade lymphoma, the most common being marginal zone B-cell lymphoma [6,9-10]. In a comparison of PCVL and PDL, there are much fewer reports of calvarial involvement and intraparenchymal invasion in PDL [11-13]. Our case had extensive invasion and growth with extra-, intra-, and cranial involvement. Taking this into consideration with the pathology findings of DLBCL, we believe the most likely diagnosis to be PCVL rather than one of dural origin, aligning with the definitions of other authors [6,14].

Our case is unique in several aspects: the patient presented with a history of relatively rapid growth and contrasted studies and angiography showed a large extensively vascular mass, with large feeding vessels, and aggressive sagittal sinus involvement. Initially, our differential diagnosis leaned towards a high-grade meningioma, though lymphoma and metastatic carcinoma were considered. The features of this mass, however, were seemingly inconsistent with meningioma, specifically osteolytic rather than hyperostotic bony invasion, and the intra-axial component (Table 1) [5,14]. Conversely, amongst the reported literature on PCVL, high-degrees of vascularity, with demonstrable feeding vascular branches are not described and there are no reports of confirmed sinus invasion [8,11]. Several reviews conflict in the predominance of osteolytic bone involvement, with some reporting it as rare and others reporting it as common as 74% [6,8,15]. In our case, there was profound bony invasion and destruction. With either PCVL or PDL, there are often findings of a “dural tail,” further supporting a possible meningioma diagnosis [9-10,13-14,16]. With PCVL being such a rare pathology, no signs of systemic disease, and the lack of improvement on systemic corticosteroids, we were reasonably confident that pathology would show a high-grade meningioma. This seemed to be further confirmed by the initial intraoperative frozen as aforementioned. The final report of DLBCL was unexpected but, upon review, consistent with the predominant pathology reported with PCVL [6,8].

**Table 1:** Comparison of the features of PCVL and meningioma PCVL: primary NHL of the cranial vault; NHL: non-Hodgkin’s lymphoma

Features	Meningioma WHO Grade 3	PCVL
Rapid Growth	Rapid compared to WHO grade 1/2 but not as fast as PCVL	Positive
Contrast enhancement on MRI	Positive	Positive
Dural tail	Positive	Positive
Osteolysis vs hyperostosis	Hyperostosis is more common	Most reports with either no bone destruction or osteolytic destruction
Vascularity	Positive arterial feeders	Typically negative for arterial feeders. This case was positive
Brain invasion	Negative	Not clear with some cases reporting invasion but most not. This case had brain invasion

On angiography, the right superficial temporal artery, the left occipital artery, and the right distal MMA were found as vascular supply to the mass, and we believe this to be the first case report to give such details on PCVL. Some authors report the findings on angiography of vascularity to be diagnostic of meningioma while the lack thereof more indicative of PCVL or PDL [17-18]. Secondary to the positive angiography, we followed the erroneous conclusion that this was likely a high-grade meningioma. These were initially considered for embolization but deferred when the patient elected for treatment in his home state. When he was eventually taken to the operating room, it was done urgently and no preoperative embolization was performed. We also believe this to be one of the first case reports with findings of aggressive invasion of the SSS in the setting of PCVL. While there are reports of venous sinus compression, to our knowledge, there is no literature describing confirmed venous sinus invasion. With significant compression upon a dural sinus, it becomes difficult, even with detailed vascular studies, to distinguish whether there is true invasion [17]. In our case, we were able to confirm intraoperatively that there was a frank invasion of the posterior third of the sagittal sinus by the mass.

We feel it is important to emphasize how closely this case resembled meningioma during the patient’s workup. His MRI showed a likely dural-based mass, there was aggressive bony destruction, and he had positive angiography with multiple feeding vessels. While typically extra-axial lesions, a review on invasive meningiomas by Brokinkel et al. found brain invasion in 5%-78%, with rates of invasion increasing with WHO grade [19]. A review by Crawford et al. on meningiomas reported that 59% showed hyperostotic involvement, 32% showed osteolytic, and 6% showed both [20]. Lastly, in a review on B-cell lymphoma of the dura and cranial vault, Galarza et al. reported that when considering a PCVL or dural lymphoma, ‘the most likely alternative entity to rule out is noncalcified meningioma for which angiography is usually diagnostic' [18]. The authors remark that the angiographic finding typical of lymphoma is that of an avascular tumor [18]. This was not the case with our patient who had extensive arterial vascular supply. With our report of this, though PCVL is such a rare pathology, there are very few features that distinguish it readily from a meningioma without a direct tissue biopsy.

Given such a low incidence and few reports, it is difficult to definitively establish a prognosis for these patients. El Asri et al. reported that of 22 cases in which the outcome was available, 13 of 16 patients followed out to 12 months were still alive [6]. In a review by Aquilina et al., of 16 patients presenting with diffuse primary NHL of the cranial vault, six were without systemic involvement at the time of diagnosis and continued to be disease-free after treatment with follow-ups ranging from five months to six years [7]. In the reports on PDL, it is postulated that given the majority of pathology is of lower-grade lymphomas, it is likely to have a lower incidence of recurrence and a better prognosis than those with higher grades. Amongst the reviews of PCVL, as well as primary bone lymphoma in general, it is reported that solitary vs. multiple osseous lesions carry a better prognosis overall [1-4,7-8]. However, no definite conclusion can be drawn given the small number of reported PCVL and PDL cases. A few reported PCVL cases have been followed out to at least five years so the long-term incidence of recurrence is not well-defined. Whether the patients had surgery or not, amongst all the reviews and reports of PCVL and PDL, almost all underwent CHOP (cyclophosphamide, doxorubicin, vincristine, and prednisone) or R-CHOP therapy followed by radiation. Our patient successfully completed six cycles of R-CHOP at this time and remains disease-free at 10 months.

## Conclusions

In summary, PCVL continues to represent a rare pathology that will elude consideration in a patient’s diagnosis. The most common errant diagnosis, after a review of the available cases, will be that of meningioma and multiple features on radiographic imaging will continue to make diagnosis difficult. Here, we report a case where PCVL had extensive arterial vascular supply, as demonstrated in angiography, which we believe to be the first reported of its kind. In instances of patients presenting with such extensive extracranial, cranial, and intracranial involvement, the diagnosis of PCVL will need to be diligently considered even in the face of a positive angiography.
